# The Relationship between Physical Activity and Plasma Glucose Level amongst Ellisras Rural Young Adult Males and Females: Ellisras Longitudinal Study

**DOI:** 10.3390/ijerph14020198

**Published:** 2017-02-16

**Authors:** Moloko Matshipi, Kotsedi Daniel Monyeki, Han Kemper

**Affiliations:** 1 Department of Physiology and Environmental Health, University of Limpopo, Polokwane 0700, South Africa; moloko.matshipi@ul.ac.za; 2 Institute for Health and Care Research (EMGO+), University Medical Center, Vreije Universiteit, 1081 HV Amsterdam, The Netherlands; hancgkemper@upcmail.nl

**Keywords:** physical activity, plasma glucose levels, prediabetes, rural young adults, South Africa

## Abstract

Unhealthy lifestyle characteristics such as low physical activity (PA) and high plasma glucose levels (PGLs) may lead to the development of type 2 diabetes mellitus in adulthood. The aim of this study was to investigate (i) the level of physical activity; (ii) the prevalence of pre-diabetes and (iii) the relationship between PA and plasma glucose level in a rural Ellisras adult population aged 18 to 28 years. A total of 713 young adults (349 males and 364 females) who took part in the Ellisras Longitudinal Study participated in the study. Fasting plasma glucose levels were analysed using Accutrend glucose meters. Physical activity data was collected using a validated questionnaire. Linear regression was used to assess the relationship between PA and pre-diabetes. The prevalence of pre-diabetes was between 45.7% and 50.2% and that of physical inactivity was 67.3% and 71.0% for males and females, respectively. There was no significant (*p* > 0.05) relationship between PA and pre-diabetes (beta = 1.016; 95% Confidence Interval from 0.352 to 2.777). The health benefits of PA increased with the increasing frequency, duration and intensity of exercise. The prevalence of pre-diabetes was found to be very high in this population. Our results suggest that greater physical activity is associated with low plasma glucose levels.

## 1. Introduction

The number of people with type 2 diabetes mellitus (T2DM) was noted to be increasing worldwide and is expected to be 592 million by the year 2035 [1,2]. Hossain et al. [3] added that this increase is closely linked to the upsurge in obesity. Furthermore, Lloyd-Jones et al. [4] note that T2DM is a risk factor of vascular disease with 65% of all deaths in people with diabetes due to cardiovascular disease. Although the improvement of the population’s knowledge and awareness of the risk factors associated with T2DM is essential to prevent future complications of the disease [5], such information appears to be limited to rural populations where modernisation and industrialisation take place [6].

Physical activity (PA), such as household, occupational, travel-related and occupational activities, plays an important role in the control of both weight and plasma glucose, thus reducing the likelihood of developing T2DM [7]. Physical activity improves insulin sensitivity, which helps with the uptake of glucose by cells [7]. Little is known about the benefits of PA on the prevention and treatment of T2DM in the rural South African population.

Preliminary results of the Ellisras Longitudinal Study (ELS) showed a decreased PA in both girls and boys over time [8]. It was also shown that T2DM did not existing in the sample population of ages three to 15 years [9]. However, the prevalence of obesity, overweight and hypertension was reported to be on the rise among the Ellisras children over time, indicating the possibility of the emergence of diabetes [10]. As the age of the ELS sample increases, the possibility of unknown cases of chronic diseases arises in the population. Currently no study has assessed the relationship between PA and blood glucose in Ellisras adults; thus, this study attempts to address the gap.

The aim of this study was, therefore, to investigate the levels of PA, the prevalence of pre-diabetes, the relationship between PA and plasma glucose level, and the risk of developing diabetes amongst Ellisras rural young adults aged 18 to 28 years.

## 2. Materials and Methods

### 2.1. Geographical Area

Ellisras, now known as Lephalale, is a deep rural area situated within the northwestern area of the Limpopo province, South Africa. About 50,000 people reside in 42 settlements [11]. These villages are approximately 70 km from the Ellisras town (23°40’S, 27°44’W), which is adjacent to the Botswana border. The Iscor coal mine and the Matimba electricity power station are the two major sources of employment for many Ellisras residents. The remaining workforce is involved in subsistence farming and cattle rearing while the minority is in education and civil service. Unemployment, poverty, and low life expectancy play a significant role in rural South African populations, and the Ellisras rural area is no exception [12].

### 2.2. Sample

Details of the ELS research design and sampling have been reported elsewhere [13]. For the purpose of this analysis, data collected in December 2014 and January 2015 are included. In total, 717 young adults (352 males and 365 females), aged 18 to 28 years, who are part of the ELS took part in the survey.

The Ethics Committee of the then University of the North, now known as University of Limpopo, granted ethical approval prior to the survey (Project Identification ID: MREC/P/204/2013: IR). Permission was granted by tribal authorities and the principals of schools. Participants read and signed informed consent.

### 2.3. Physical Activity

The International Physical Activity Questionnaire [14] was found to be reliable and valid. The questionnaire was used to record data on work, leisure, and travel activities both on week days and weekends. The score for these activities were attained by multiplication of the number of reported activities with the number of days engaged in those activities per week. The total PA score was calculated as a summation of the types of activities for each participant. The duration of these periods was added up to give a mean duration for moderate to vigorous physical activities during weekdays and weekends. Metabolic equivalents (METs) express the energy cost of physical activities as a multiple of the resting metabolic rate and yield a score in MET-minutes. MET-minutes were obtained by multiplying the MET score (8 for vigorous and 4 for moderate activity) by the minutes performed [15].

The principal investigator with the help of Ellisras local teachers translated the questionnaire from English to the two local spoken languages (Northern Sotho and Setswana) and then translated back to English. The back translation to English showed no disparity with the Northern Sotho and Tswana languages.

The Senior Northern Sotho and Tswana speaking Physiology and Environmental Health department students of the University of Limpopo who were specifically trained for using this questionnaire, interviewed the participants at local schools/halls; with each interview lasting for about 30 min.

### 2.4. Fasting Plasma Glucose

Participants were asked to fast for 8–10 h before blood collection. All collections were done by a registered nurse at a local schools/halls. Fasting venous blood samples were collected in the morning [16]. Fasting blood samples were collected into 4 mL grey top vacutainer tubes (vacutainer BD^TM^) containing sodium fluoride and oxalate. Samples were then placed in a cooler box with ice (0–8 °C) on site. At the laboratory, fasting blood samples were centrifuged to obtain plasma at 2500 rpm for 15 min. Clotted and hemolysed samples were discarded. Plasma was stored at −80 °C for later analysis. Measurements were done in the University of Limpopo’s Medical Sciences laboratory using access supplied by Beckman Coulter.

### 2.5. Dietary Intake

Diet was measured using the 24 h recall method, which is a valid method to determine group dietary intakes [17]. Senior Northern Sotho speaking dietetic students of the University of Limpopo, specifically trained in using socioeconomic questionnaire and the 24 h recall method, interviewed the participants at local schools on the dietary intake over the previous 24 h. For each participant, the interview took place on one weekday and on one weekend day in order. An average of two days 24 h dietary intake was then made for each participant. Estimated portion sizes of foods consumed were recorded using a pre-tested questionnaire and food models simulating average portions of local foods [18,19]. Dietary data was analysed using local food tables and software [18] and compared with recommended intake [20].

### 2.6. Employment

Information was collected regarding employment by means of a questionnaire. Participants were asked to answer ‘yes’ if they are employed and ‘no’ if not.

### 2.7. Statistical Analysis

Descriptive statistics of the three PA domains (work, leisure, travel) together with the plasma glucose concentration were reported. All participants were classified as inactive, minimally active, or sufficiently active according to the International Physical Activity Questionnaire (IPAQ) [14] cut-off point that categorises physical activity as inactive (<600 MET-min/week), moderately activity (600–1500 MET-min/week) and sufficiently active (≥1500 MET-min/week). The American Diabetes Association [21] cut-off points were used to define pre-diabetes as plasma glucose level between 5.6 and 6.9 mmol/L (inclusive). Chi-square test was used to compare two or more sets of nominal data that have been arranged into categories by frequency counts of large samples, while the Fisher’s exact test was used when the expected cell frequencies were smaller (less than five). A linear regression model was used for assessing the relationship between physical activity levels and plasma glucose unadjusted and adjusted for family history of diabetes, age, gender and dietary intake. Logistic regression was used to assess the risk of developing pre-diabetes for unadjusted and adjusted for family history of diabetes, age, gender and dietary intake. All the statistical analyses were done using SPSS software (version 23, IBM, Armonk, NY, USA). Statistical significance was set at *p* < 0.05.

## 3. Results

A high unemployment rate (68.2%) was reported amongst the ELS sample aged between 18 to 28 years. Table 1 shows descriptive statistics for MET-min/week in the work, leisure and travel domains and the proportions of total physical activity with the corresponding plasma glucose concentrations of each age group. Males (an average of 783.05 MET-min/week) were significantly (*p* < 0.05) more active than females (an average of 777.52 MET-min/week). Plasma glucose concentrations were significantly (*p* < 0.05) higher for females (5.66 mmol/L) compared to those of males (5.53 mmol/L).

Table 2 shows the percentage distribution across PA categories of inactive, minimally and sufficiently active based on MET-min/week. The majority of the participants (males = 67.3%; females = 71.0%) could be classified as inactive (i.e., less than 600 MET-min/week). Of the remaining participants, 9.4% and 10.1% males and females were, respectively, classified as minimally active (i.e., PA levels between 600 and 1500 MET-min/week) and 23.3% males and 18.9% females were sufficiently active (at least 1500 MET-min/week).

Table 3 shows the descriptive statistics for the 24 h dietary intake recall by gender of Ellisras rural young adults aged 18 to 28 years. The intake of carbohydrates was significantly (*p* < 0.05) higher for males (109.9) compared to females (105.3). Fat intake was significantly (*p* < 0.05) higher for females (21.0 g) compared to males (16.9 g).

**Figure 1:**
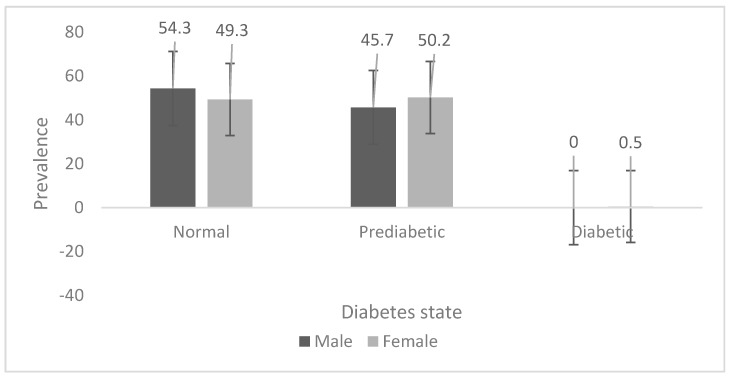
Prevalence of pre-diabetes and diabetes.

The prevalence of pre-diabetes is shown in Figure 1 for the Ellisras rural young adults aged 18 to 28 years. The prevalence of pre-diabetes was significantly (*p* < 0.05) higher in females (50.2%) than in males (45.7%). Only 0.5% of females had diabetes whilst no males had diabetes.

Linear regression showed a significant (*p* < 0.05) relationship between PA and pre-diabetes for the work category both unadjusted and adjusted for family history of diabetes, age, gender and dietary intake (beta = 0.004, 95% CI: 0.003 to 0.005); however, no significant relationship was shown between the total physical activity and pre-diabetes.

Logistic regression showed no significant relationship between PA and pre-diabetes, both unadjusted and adjusted for family history, age, gender and dietary intake (beta = 0.016, 95% CI: 0.364 to 2.832).

## 4. Discussion

The aim of this study was to investigate the relationship between PA and the plasma glucose level amongst Ellisras rural young adults aged 18 to 28 years. A significant positive relationship between physical activity and glucose level was recorded in the current study. The results of this study showed that there was an overall low level of physical activity amongst Ellisras females as compared to males. It was also observed that males spend generally more time doing work-related activities than females. Micklesfield et al. [22] found similar results in adolescents of the Mpumalanga Province of South Africa. Our study supports an assertion by Amosun et al. [23] that over a third of South African children are inactive (67.3% males and 71.0% females in this study). Furthermore, the low levels of physical activity in this population may be attributed to factors such as poverty [24] and environmental factors such as crime, lack of security, time constraints, inability to afford exercise equipment and lack of recreational facilities [25].

The overall plasma glucose concentrations for females (average 5.67 mmol/L) were significantly higher than those of males (average 5.51 mmol/L). This is similar to what McCollum et al. [26] found, although their study was based on an older population, where women were significantly older than men, and had less education and lower incomes (which may lead to the consumption of unbalanced diets).

In the current study the prevalence of pre-diabetes was high (males = 45.7%; females = 50.2%) compared to the 25% (for both genders each) found by Okafor [27] in Nigeria. This difference could be due to the high levels of inactivity (males = 67.3%; females = 71.0%) and high family history of diabetes (males = 21.6%; females = 28.5%) in this population. The high prevalence and long-term implications on health make diabetes a major concern for developing countries [28].

The logistic regression showed no significant relationship between PA and pre-diabetes. This was different from the findings by Hu et al. [29] who studied 70,102 nurses in the United States. The contradiction may be due to the large difference in sample size between the two studies, with the later study having probably assumed more than 80% power at the 0.05 significance level.

This study is cross-sectional; thus, longitudinal data needs to be collected in order to conclude on the causal relationship between PA and T2DM in this population. Furthermore, diabetes is a multi-faceted disease with numerous risk factors; physical activity alone is not enough to predict the possibility of developing diabetes, as reported in other studies [23,24]. However, physical activity plays an important role in the control of both body weight and plasma glucose, thus reducing the likelihood of developing T2DM [7]. Recall of the time spent doing physical activity could introduce bias in the study, particularly in Sub-Saharan Africa, given the illiteracy level [8]. However, well-trained fieldworkers were reported to provide accurate information in South Africa and Nigeria [30] as was the case in the current study.

## 5. Conclusions

The prevalence of pre-diabetes (46%) was high in this group of Ellisras young adults aged between 18 to 28 years. Our results suggested that physical activity was associated with low plasma glucose levels, although the logistic regression showed no relationship between physical activity and pre-diabetes. Further studies are needed to investigate the development of biological and behavioural risk factors for diabetes in rural South African populations over time.

## Figures and Tables

**Table 1:** Descriptive statistics for MET-min (metabolic equivalent minutes) per week in work, leisure and travel domains and proportions of total physical activity with corresponding plasma glucose concentrations of each age group for 717 ELS young adults aged 18 to 28 years.

Age (Mean ± SD)	Males (n = 352)						Females (n = 365)					
	n	Mean (SD) MET-Min/Week				Plasma Glucose Concentration (mmol/L)	n	Mean (SD) MET-Min/Week				Plasma Glucose Concentration (mmol/L)
		Work	Travel	Leisure	Total			Work	Travel	Leisure	Total	
20.99 ± 0.7	92	40.00 * (69.66)	480.44 (153.06)	235.83 (93.18)	756.27 (276.13)	5.57 (0.97)	77	31.17 * (63.78)	457.77 (183.89)	265.86 (492.07)	754.80 (300.91)	5.50 (0.88)
23.07 ± 0.6	104	58.46 * (77.42)	495.22 (190.56)	258.20 (151.06)	811.88 (299.22)	5.63 (1.005)	107	55.33 * (76.46)	556.91 (380.53)	267.15 (422.79)	879.39 (478.12)	5.70 (0.867)
24.95 ± 0.6	109	57.25 (77.05)	485.36 (175.88)	261.27 (104.61)	803.88 (305.65)	5.49 (0.94)	128	45.00 (72.22)	468.38 (157.14)	207.80 (522.76)	721.18 (235.28)	5.69 (2.19)
26.61 ± 0.4	47	68.09 (79.96)	430.98 (156.15)	261.1 (132.15)	760.17 (264.38)	5.34 (0.737)	53	57.36 (77.46)	439.85 (154.72)	257.51 (568.56)	754.72 (272.55)	5.77 (0.89)

METs are multiples of the resting metabolic rate and yield a score in MET-minutes, which is computed by multiplying the MET score (8 for vigorous and 4 for metabolic activity and travel-related walking/cycling) by the minutes performed. * *p* < 0.05.

**Table 2:** Percentage distribution of employment history, family history of diabetes, and physical activity categories of inactive, minimally and sufficiently active for ELS young adults aged 18 to 28 years based on MET-min/week.

Category		Males (n = 352)	Females (n = 365)
		% (n)	% (n)
Physical activity level	Inactive 0–600 MET-min/week	67.3 (237)	71.0 (259)
	Minimally active 600–1500 MET-min/week	9.4 (33)	10.1 (37)
	Sufficiently active ≥1500 MET-min/week	23.3 (82)	18.9 (69)
Employment	Yes I am employed	34.7 (122)	29.3 (107)
	No I am not employed	65.3 (230)	70.7 (258)
Family history of diabetes	Yes there is family history of diabetes	21.6 (76)	28.5 (104)
	No family history of diabetes	78.4 (276)	71.5 (261)

METs are multiples of the resting metabolic rate and yield a score in MET-min/week, which is computed by multiplying the MET score (8 for vigorous and 4 for metabolic activity and travel-related walking/cycling) by the minutes performed.

**Table 3:** Descriptive statistics for 24 h recall dietary intake by gender of Ellisras rural youth aged 18–30 years (n = 728).

Variables	Males	Females
	Mean (SD)	Mean (SD)
Energy	3287.0 (1629.3, 5275.3)	3689.0 (1357.0, 5516.0)
Carbohydrates	109.9 (52.1, 173.3) *	105.3 (35.2, 175.2) *
Total protein	40.7 (13.8, 72.7)	31.3 (8.3, 53.2) *
Total fat	16.9 (4.7, 34.4) *	21.0 (5.3, 43.1)
Total sugar	0.8 (0.0, 6.35)	2.0 (0.0, 8.5)
Cholesterol (mg)	65.5 (1.0, 145.0)	69.0 (3.0, 176.0)
Fibre	4.5 (1.3, 9.8)	5.3 (0.6, 9.4)
Iron	3.2 (1.5, 5.6)	3.9 (1.2, 6.5)
Vitamin A	34.5 (1.0, 132.0)	40.0 (0.0, 176.0)
Vitamin E	1.2 (0.3, 3.9)	1.4 (0.2, 4.7)

SD: standard deviation; * = Exceeds RDA: recommended dietary allowance: Recommended Daily Allowance according to the Food and Agriculture Organization [20].

## Abbreviations

The following abbreviations are used in this manuscript:

ELS	Ellisras Longitudinal Study
MET	Metabolic equivalent
PA	Physical activity
CVD	Cardiovascular diseases
PGLs	Plasma glucose levels
RDA	Recommended daily allowance
SPSS	Statistical Package for Social Sciences
T2DM	Type 2 diabetes mellitus
